# The genesis of Brexit in the UK: outline of a multi-field model

**DOI:** 10.1007/s11186-022-09483-3

**Published:** 2022-05-17

**Authors:** Will Atkinson

**Affiliations:** School of Sociology, Politics and International Studies, University of Bristol, Bristol, BS8 1TU UK

**Keywords:** Brexit, Class, Ethnicity, Fields, Habitus, Politics

## Abstract

This paper outlines a sociological model of the conditions of possibility of the UK’s decision to withdraw from the European Union in 2016. Drawing on the conceptual tools of Pierre Bourdieu and those inspired by him, it synthesises and goes beyond the partial and fragmentary accounts offered so far to offer a more comprehensive narrative implicating the interrelation of multiple fields, with agents’ evolving strategies within the different fields being the major fulcra. To be specific, the conditions of possibility for the referendum result were provided by mutations within the global field of nation states ricocheting through the UK’s political field, ethno-racial field and class structure.

## Introduction

The UK’s withdrawal from the European Union (EU) is one of the most profound events in the recent history of the country, igniting a period of significant political turmoil lasting from the initial referendum result in 2016 to today. It was also one of the most shocking for the EU, and its remaining constituent members, setting a new precedent for secession counter to the general trend of enlargement. Unsurprisingly, therefore, a gush of analyses has flowed forth from think tanks, journalists and academics across a dizzying variety of disciplines, all eager to work out exactly how withdrawal from the EU, aka ‘Brexit’, will impact on international relations and domestic politics and how a majority vote for it came about in the first place. On the second of these questions, which is more the intellectual territory of the sociologist, a plethora of theories has been offered, some of them based on research, some of them seemingly based on little more than political belief, but all of them partial in one way or another.

The aim of this paper is to sketch a working sociological model of the genesis of Brexit that integrates and goes beyond those provided by others. It is not intended to be a definitive statement or exhaustive analysis, which would be impossible within the confines of a single paper, so much as what Pierre Bourdieu would call a ‘construction of the object’: a reasoned, logical yet selective vision of the phenomenon in question aimed at identifying the relevant social structures, their interrelations and their salient transformations furnishing its principal conditions of possibility, informed by observed reality yet also generating hypotheses for follow-on research (Bourdieu et al., 1991). The task is doubtless complicated by the fact that the object of investigation is a moving target: the full meaning of Brexit is yet to unfold, its effects still unfurling and its magnitude or reversibility yet to be determined. Yet our focus is on what has passed – the social history of a specific past event with evident consequences for what came after – making it as much amenable to social scientific investigation as the last general election, the French Revolution or, indeed, the Battle of Marathon.

The core components of the model are the conceptual tools provided by Bourdieu, specifically the notions of capital, habitus and field. These allow us to synthesise and reframe existing research and documentary evidence and, ultimately, to suggest that Brexit was, to use Bourdieu’s phrase, the product of a shifting *feel for the game*. Yet this practical sense existed at multiple interpenetrating structural layers that need to be unpicked. These are the layers of *global* relations of power, national *political* struggles and national *class* and *ethno-racial* structures. It thus becomes necessary to go beyond Bourdieu’s strict blueprint for sociological analysis to take the interrelations between fields, and the positioning of singular agents in multiple fields, more seriously. The ultimate argument will be that strategies played out in global and political struggles appeared to have unintended consequences for class and ethno-racial relations which then, in turn, produced transformations within the national political field providing the conditions of possibility of Brexit.

## Elements of the model

Most analyses and commentaries on the genesis of Brexit tend to agree that long-run trends in the social and political fabric of the UK are fundamental (see e.g. Evans & Tilley, 2017; Evans & Menon, 2017; Clarke et al., 2017; Eatwell & Goodwin, 2018; Dorling & Tomlinson, 2019; Norris & Inglehart, 2019; Piketty, 2020). On the one hand, deindustrialisation, the rise of the service sector, the decline of trade unions, the expansion of higher educational provision, the feminisation of the workforce, globalisation, migration, neoliberal deregulation and deepening economic inequality from the 1970s are all, say most, critical parts of the puzzle. On the other hand, the headline story of political transformation leading up to Brexit is the electoral surge of nationalist populism, and this is, itself, said to be a reaction against the increasing liberalism and cosmopolitanism of electorates and party politics in the wake of affluence, globalisation and the diffusion of higher education. Symptomatic of this reactionary national populism was the growing prominence of the UK Independence Party (UKIP), led by former stockbroker Nigel Farage. Far from being a British peculiarity, however, nationalist populism and the pervasive liberalism it challenged are said to be global phenomena, responsible for the electoral victories of Viktor Orbán and Donald Trump amongst others, though only in Britain has such a long-term critical rupture as departure from the European Union been the result.

The studies cited tend to focus on one aspect or another of the story, the most common of which is undoubtedly the relationship between social class and party politics. Many of these adopt, more or less explicitly, the ‘imaginary anthropology’ of rational choice theory (Bourdieu, 2005), as befits the current orthodoxy of political science and political sociology, but otherwise they are riven by apparent dissension. Some say the rise of populism marks the *demise* of class politics and the increasing prominence, instead, of education as a socio-political cleavage (e.g. Eatwell & Goodwin, 2018), while others claim it merely marks a *reinvention* of class politics (e.g. Evans and Tilley, 2017). Either way there is a tendency to leave core structural contexts for the changing class-politics link unexplained, including deindustrialisation, the mutating state of international relations, the legacy of empire and transformation of ethno-racial relations in the UK. Those that do emphasise these factors, however (e.g. Seidelr, 2018; Bhambra, 2017, 2018; Dorling & Tomlinson, 2019), often in explicit opposition to mainstream narratives, tend to either marginalise class or treat it very vaguely – often in quasi-Marxist fashion or using rudimentary occupational classifications – and to lack a meaningful grasp of the nexus between social structure and individual practice to compete with the reliance on rational choice models among others.

Bourdieu’s tools, in contrast, can be used to provide not just a comprehensive and integrative account of how Brexit came to pass, combining the national and international, the cultural and the economic and class and race/ethnicity, but one freed from the distortive illusions of utilitarianism. The starting point is the notion of field, understood as a system of struggle and domination between a set of agents revolving around a relatively autonomous form of misrecognition – that is, an arbitrary characteristic or property misperceived as making its bearer inherently worthy or legitimate. These forms of misrecognition Bourdieu famously called ‘capitals’, and they are relatively autonomous when they cannot be reduced down to any other capital. Fields are typically multidimensional because there is usually more than one kind of capital in play, with at least one of them being specific to that field alone. So, for example, the literary field is structured according to two forms of capital: the economic capital of profits from sales and the ‘specific’ capital of esteem from fellow writers and critics revolving around artistic merit (Bourdieu, 1993, 1996a). There is thus a polarity distinguishing those who are more and less successful, or ‘consecrated’, within the field, but also a polarity distinguishing those whose success derives primarily from sales on the mass market and those whose reputation is based instead on peer recognition – and, of course, there is plenty of room in between for agents with different combinations and amounts of capital.

Contrary to common interpretations of Bourdieu’s concepts as being somewhat static and ahistorical (e.g. Connell, 1983; Jenkins, 2002), the notion of field is inherently temporal (Atkinson, 2019). This applies even to the most supposedly deterministic of related ideas, the habitus, defined as the agent’s system of dispositions and schemes of perception inclining them towards specific practices and produced out of adaptation to position within the field. The habitus is nothing more than the individual’s anticipations of the future – their sense of what is possible, feasible and desirable in the struggle for capital given the capital they possess and the recent history of the field. This is the ‘feel for the game’ that Bourdieu (1990a) liked to talk about, the baseline for people’s strategies and tactics within the field to accumulate capital (i.e. seek worth), and ultimately it is grounded in the phenomenology of time, specifically the expectation of the immediately forthcoming inscribed in present perception given past experience that Husserl (1991) labelled ‘protention’.

Part of the history of the field feeding into individual habitus is the history of the field’s movements - of who is falling or rising within the field, who are newcomers and the old guard - and how one sits in relation to that. Being part of an embattled but dominant old-guard can incline one to a rather conservative view and strategies aimed at maintaining the status quo. Those on the rise might work within orthodox parameters, and become successors, but they might instead intuit that the most feasible and desirable strategy for them is to try and *subvert* the game – to change the definition of what is valuable and overthrow the old guard entirely. This can culminate in moments of *revolution* within fields, as everything changes, and sometimes this might be accompanied by a period of anomie – uncertainty, rapid rises and falls and so on – before the field settles back into a more stable structure (Bourdieu, 1996a, 2017; see also Steinmetz, 2018; Fowler, 2020).

**Fig. 1:**
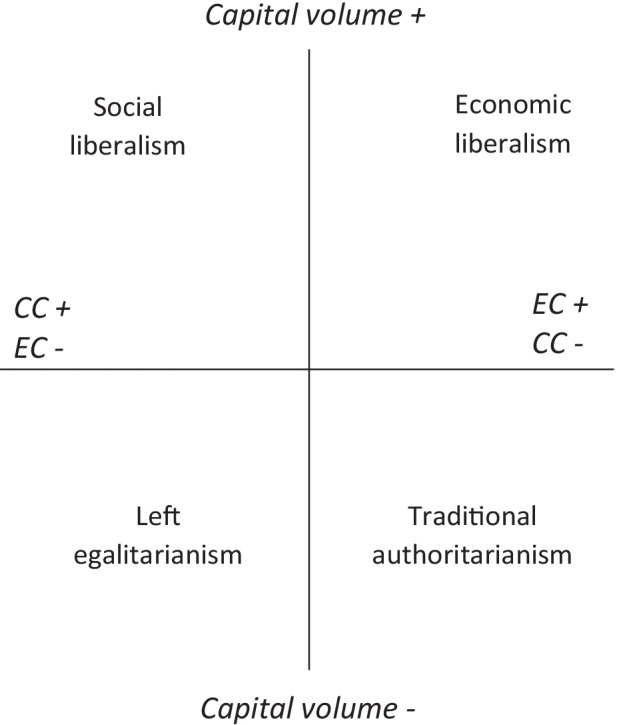
Model of the typical relationship between the social space and political position-taking

Contemporary social orders are characterised by a plurality of fields that interrelate in a certain way. At the centre is the specific field that Bourdieu (1984) called the social space, or, less often, the ‘field of classes’. This is the major field of a national social order, defined by the key forms of misrecognition prevailing there and implicating all agents in one way or another. In advanced capitalist social orders its structure is given by the distribution of three capitals: economic capital (money and wealth), social capital (connections and memberships) and cultural capital (valued modes of knowledge and language concretised in educational qualifications). Dominant, intermediate and dominated classes are defined by their volume of capital as a whole, but – contrary to those perspectives that cleave education from class – class fractions are defined by their composition of capital, distinguishing those characterised largely by economic capital (business owners, skilled workers, etc.) from those possessing a greater balance of cultural capital (artists, teachers, intellectuals, etc.). Position within the social space generates a sense of place and of possibilities within it, i.e. the feel for the game, and, tied up with that, a specific *ethos* manifesting in position-takings on political or moral matters – redistribution, gender roles, migration and so on. These position-takings form a space of their own homologous with the social space – as research has shown time and again (Bourdieu, 1984; Harrits et al., 2010; Flemmen, 2014; Flemmen and Haakstaad, 2018; Atkinson, 2017), economic capital goes hand-in-glove with economic liberalism while cultural capital corresponds with social liberalism, and those lacking both are inclined to be put off politics altogether (Fig. 1).

Within the social space there is the ‘field of power’ – the field of struggle between those at the top of the class structure to define the primary form of misrecognition in a social order, that is, economic or cultural capital (Bourdieu & Wacquant, 1993; Bourdieu, 1996b). Yet this field is also split into a number of constituent subfields with their own relatively autonomous species of capital that condense and refract larger struggles. There is the field of business (or ‘economic field’), the field of academia, the state field (or ‘bureaucratic field’), and there can even be sub-subfields if a relatively autonomous form of capital emerges (see Bourdieu, 1988, 2005, 2014). One field of particular relevance here is the *political* field, understood as the field of struggle between agents contending to represent the worldview and interests of specific sections of the population (Bourdieu, 1991). Elected and unelected politicians, trade union leaders and heads of campaigning organisations and lobby groups are its core members, with the specific stake in the field being the number and resources of supporters one can rally and, with that, degree of leverage over the machinery of government (i.e. the bureaucratic field). Part of this struggle – linking back to the field of classes – is the formulation of an explicit ‘party line’ appealing to the ethos of different sections of the social space, one result of which is a structural correspondence between the social space and affiliation (identification, membership, voting) with specific political parties or organisations.

The political field is structured, according to Bourdieu (1991), by possession of two forms of political capital: *bureaucratic* political capital, in the form of officially recognised titles and positions within political parties or organisations, and *personal* political capital, perceived as ‘charisma’, which is little more than the translation of outside capitals, like economic or cultural capital, into a mode of misrecognition when speaking on political matters (as symbols of one’s ‘competence’, ‘shrewdness’ or ‘intelligence’). Moments of revolution in the field often come, as they do in the religious field, when a new entrant to the field, typically harnessing personal political capital, articulates the ethos of a large section of the social space that was previously neglected or distorted by the established players.

To this picture we can add two fields that Bourdieu did not consider but which others after him have posited with compelling logic and evidence. The first of these is the ethno-racial or ethno-national field (see esp. Hage, 1998; Emirbayer & Desmond, 2015; Atkinson, 2020a). This is the national-level field of difference and domination, or misrecognition, orienting around perceptions of ‘origins’, associated symbols and practices (dress, language, skin colour) and the different levels of worth accorded to them. Whether it is structured primarily around notions of ‘race’ or ‘nationality’ or is more mixed and ambiguous, and the degree of autonomy it enjoys from the social space, are historical, empirical questions for each ethno-racial/national field, though class-specific versions of ‘whiteness’ have long functioned as sources of symbolic capital in most Western social orders. Wacquant (2008), for example, is firmly of the view that ‘race’ is the prime organising principle of difference in the US context – as are Emirbayer and Desmond (2015) – but that ethnicity or nationality plays a greater role in Europe (a view extended implicitly to Australia by Hage, 1998, and Tabar et al., 2010).^1^

Second, there is what Julian Go (2003) has called the ‘global field’, or what others have referred to as a ‘field (or space) of states’ (Schmitz & Witte, 2020; Atkinson, 2020a, 2020b, 2022; see also Nexon & Neumann, 2018). This is the field of struggle and domination between *nation states*, or rather the key agents within national bureaucratic fields acting as agents of the nation state in international politics. This concept serves as a reconceptualization of international relations and the practices it generates (war, annexation, trade deals, etc.). Out go the dominant images of the unitary state as either (i) a rational actor aiming to maximise its security through economic and military might or alliance, as in ‘realist’ versions of international relations theory, or (ii) a node in a network of unbalanced economic interdependencies, as in world-systems theory. In comes a model of delegated state actors, contemporaneously engaged in their own domestic struggles, pursuing strategies in a global game for multiple forms of capital delivering symbolic power to their nation on the world stage. The major capitals in play are economic capital (national revenue and purchasing power), cultural capital (in the form of cultural exports and facility with the dominant language), technical capital (in the form of valued workforce skills in the global division of labour), political capital (voice in international affairs, especially via membership of transnational political bodies, but also indicators of political legitimacy such as anti-corruption) and military capital (size and capacity of armed forces).^2^

Bourdieu was famously of the view that analysis of a field, as the ‘focus of the research operations’, should be sensitive to its interrelationship with other fields, particularly the field of power (Bourdieu and Wacquant, 1992: 104–7). His general practice, however, was to focus on the structure and transformations of a single field and keep other fields, or mutations in the larger social space, in the background, as explanans rather than explananda – this is certainly how he treats contextual transformations of the social space underlying Manet’s or Heidegger’s symbolic revolutions in their respective fields (Bourdieu, 1990b, 2017). Yet Brexit is a thoroughly trans-field phenomenon. It implicates agents who are themselves situated within multiple fields, balancing multiple interests and feels for a game, like the national politician also delegated to act in the field of nation states (see Atkinson, 2016; Schmitz et al., 2017). And it implicates a dialectical interplay between multiple fields, which, concretely, takes the form of myriad manifestations of the effects of strategies within one field (ideas, goods, events, etc.), actualised in circuits of spatiotemporal movement of and exchanges between people and things, being translated or ‘restructured’ into the logic and stakes of another field (Bourdieu and Wacquant, 1992: 105). Sometimes these congeal into specific interdependencies between fields, like the supply/demand relation between the political field and the social space, or the reliance of many fields on the state for their existence (Bourdieu, 2014), or the emergence of trans-field ‘worlds’ of reciprocal interaction and exchange of the kinds studied by interactionists (Atkinson, 2020a; see also Krause, 2017). Our task, in any case, is to limn in broad terms the inter-field articulations providing the conditions of possibility of Brexit on the understanding that the details of intra-field struggles have been or will be filled in elsewhere.

## The field of nation states, the political field and the ethno-racial field

The starting point is the field of nation states. More particularly, the key here is the evolution of the field of nation states through the 20^th^ Century and the specific trajectory of the UK within it. The trajectory is one of relative decline, i.e. loss of economic, military and political capital, and the facts here are well known: economic devastation after the Second World War with loss of shipping and exports, depletion of gold reserves and the costs of rebuilding (dependent on the Marshall Plan and sales of overseas assets); further diminution of economic and military capital with the loss of empire as lands and subjects seceded and trading rights dissipated; and loss of influence on the world stage signalled by the Bretton Woods agreement, in which the US dollar became paramount, and perhaps most forcefully by the failed effort to maintain control of the Suez canal in 1956 (Peden, 2012). The US assumed the dominant position in the field while the USSR busily pursued numerous strategies to accumulate economic and military capital.

By the 1960s, British state actors sensed the shifting (im)possibilities for the UK in this new global context and began to formulate and pursue several *conservation strategies*, or what might really be defined as compensation or conversion strategies. One of these was the effort by the governing Labour Party under Harold Wilson to make up for the loss of global economic and political capital by cultivating *technical capital* through the ‘white heat’ of workplace modernisation.^3^ In his famous speech of 1963, Wilson declared that ‘the influence of Britain’ can no longer depend upon ‘nostalgic illusions’ or ‘accumulated reserves’, that the country must ‘adapt itself to different conditions’, ‘earn’ its influence and avoid becoming ‘a stagnant backwater, pitied and condemned by others’ by embracing technology and opening up higher education to greater swathes of the young population. The Robbins Report on higher education, commissioned and published by the government at the same time, reinforced the proposal to dramatically expand tertiary education, including technological training, so as to create a pool of workers capable of turbo-charging the economy.

Another conservation strategy, dovetailing with the interests of the masters of the economic field (as represented by the Confederation of British Industry), was membership of the European Economic Community (EEC). This was conceived as a means to bolster not just national *economic capital* but also *symbolic power* through favourable trade conditions with dominant agents in the field of nation states, since the former colonies and the Commonwealth returned fewer and fewer dividends. ‘Our influence in world monetary and trade discussions would be destroyed’ without entering into the EEC, declared the Conservative Prime Minister, Ted Heath, in parliament, as “[t]hese questions would be settled by the United States, the European Community and Japan’.^4^ Yet it was contested both within the field of nation states – Charles de Gaulle’s opposition to Britain’s entry in a bid to augment France’s position being the most prominent manifestation – and the British political field, as leftist Labour politicians (e.g. Barbara Castle, Tony Benn) viewed the regulations of the EEC as anti-socialist and right-wing Conservatives (e.g. Enoch Powell) portended a loss of sovereignty. Accession came in 1973, and Wilson oversaw a referendum confirming the majority preference for staying in the EEC under renegotiated terms.

As the 1970s marched on, however, British GDP and economic growth lagged behind that of its European rivals. Having been the leading economy of Europe as late as 1965, in 1970 the UK’s GDP was 88 percent that of France and 61 percent that of Germany; by 1975 it was two-thirds the GDP of France and less than half the GDP of Germany (figures were about the same in 1979).^5^ This was exacerbated by the oil embargo of 1973 and the economic devastation – namely, stagflation and unemployment – it wreaked upon the West. A sense of both *crisis* and *decline of the UK* *within the field of nation states* became widespread among agents in the British political field, and the broader population too (Tomlinson, 2000; Turner, 2008). The subsequent adoption of neoliberal principles and a policy of facilitating deindustrialisation in this context was not merely a capitalist conspiracy (as for Marxists like Harvey, 2005) or a simple ‘rational choice’ in pursuit of party power (on the famous model of Downs, 1957) but determined by multiple interlocking factors. The resonance of neoliberalism with Margaret Thatcher’s petit-bourgeois class ethos and a battle within the British political field to undermine the strength of the trade unions were among them (see Atkinson et al., 2012; Atkinson, 2017), but adaptation to the field of nation states was also key. Facilitating the switch from industry to services, knowledge production and finance, and the economic deregulation and expansion of higher education to foster ‘human capital’ (i.e. technical capital) that went with it, were further tactics of the *ongoing conservation strategy* in the field of nation states.^6^ The uptake of neoliberalism and deindustrialisation were, therefore, *multiple plays*, or what might be called ‘alloy strategies’, involving myriad agents implicated in more than one field.

The language of governing Conservative Party manifestos in the 1980s is indicative of this multi-field strategy.^7^ In 1983, for example, there were lamentations that ‘British industry was uncompetitive, over-taxed, over-regulated and over-manned’ before Thatcher came to power four years earlier, making Britain ‘one of the least efficient and most over-manned of industrialised nations’, and only the deregulation and thinning of industrial workforces the Party favoured had begun to turn things around. At the same time, the 1983 manifesto claimed that traditional industries were ‘being transformed by new technology’ (e.g. the microchip), leading to a ‘rapid rise in unemployment in almost every Western country’. ‘The truth’, the text continued, ‘is that unemployment, in Britain as in other countries, can be checked and then reduced only by steadily and patiently rebuilding the economy so that it produces the goods and services which people want to buy, at prices they can afford’ – a move apparently being blocked by the trade unions. The manifesto thus pledged a package of support for new technologies via, amongst other things, science parks, promotion of information technology in schools and the reorientation of higher education toward technology, science and engineering. This seeming continuation of Wilson’s ‘white heat’ directive, however, was paired with explicit devotion to encouraging growth in the sports, recreation and tourism sectors as sources of jobs and economic growth – i.e. lower-level services (where unionism was weak).

By 1987, although the Conservative manifesto still bellowed that ‘Nothing would destroy whole industries more effectively than a return to the overmanning and restrictive practices of the 1970s’ and that ‘High unemployment is one of the most intractable problems facing all Western industrialised countries’, the focus on new technology was less central. Instead, the manifesto hailed its encouragement of ‘growth in those crucial areas of new enterprise which provide the foundation for the jobs of the future’, especially ‘the expanding service sector – particularly tourism and leisure’. Additionally, the manifesto claimed ‘we must meet the nation's demand for highly qualified manpower to compete in international markets’ by expanding provision of higher education. All of this indicates orientation toward the interplay of mutations in the domestic social space, in the form of changing industries, unemployment rates and diffusion of cultural capital, *and* the UK’s position and possibilities in the field of nation states (in the form of ‘competitiveness’), as well as the expectation that deregulation, nourishing higher education and encouraging the service sector will solve *all* problems.

Later versions of the neoliberal orthodoxy within the British political field, particularly that championed by New Labour under Tony Blair (on which more later), put increasing emphasis on the necessity of immigration to economic growth – not just as a means of attracting the ‘best and brightest’ to its new industries but also to fill the lowest-paid and lowest-skilled service-sector positions abandoned by white Britons expecting more (Somerville, 2007). The result was a profound transformation of ethno-racial/national relations. More specifically, there is everything to suggest that the field of ethnic struggle in the UK became structured less prominently around classifications of *race* and more conspicuously around classifications of *nationality*, even if it inevitably retained racialised features. After all, post-war Britain was characterised by severe tensions articulated explicitly in terms of ‘race’. As yet another tactic to rebuild the labour force and economic growth after the Second World War, the British government had encouraged and sponsored the migration of workers from (former) colonies. Since these workers – including the Windrush generation – were defined as subjects of the British crown, differences of nationality played little part in classifications of difference; instead it was ‘race’ – anchored in skin colour – which was the defining principle of judgement and with which corresponding differences of language, accent, dress, music, food and so on were associated and articulated (cf. Bhambra, 2017, 2018). Urban concentration and segregation, delegated spokespeople and interest groups, discrimination and open conflict were all officially defined and labelled – in law, in politics, in academia, in the media – in terms of ‘race relations’ or ‘race riots’ right up until the 1980s (CCCS, 1982; Rex & Tomlinson, 1979). The perceptual black/white binary undoubtedly operated as a baseline organising principle, a yardstick for measuring relative worth, often obscuring or de-emphasising internal differentiation of both the white and non-white populations, even in academic analyses (see Anthias & Yuval-Davis, 1992).

The political emphasis on immigration in the 1990s was facilitated by transformations of the EEC in the meantime from a trade-based organisation to a proto-state encouraging freedom of internal movement, i.e. the European Union (EU). Conceptually speaking, what is described as ‘the EU’ is an interpenetration of a transnational *political* field and a transnational *bureaucratic* field embedded within a transnational *field of power*.^8^ Those fields are defined by the same species of capitals as equivalent fields at the national level, but agents are also polarised according to whether their powers reside primarily within national or transnational institutions and, in the transnational political field, their capital ‘weighted’ according to the position of the member state they represent within the field of nation states. Riven by internal struggles as the EU fields may be, however, their outcomes – and their very existence – have constituted steps toward generating a *unified pan-European social space*. Just as regional rulers once generated *national* social spaces by unifying economic and cultural conditions across territories (Bourdieu, 2014; Atkinson, 2022), so the EU has operated to equivalate economic capital (the Euro) and cultural capital (the Bologna system, promoting pan-European values, etc.). The rationale was to bolster members states’ economic performance and political stability vis-à-vis competitors in the field of nation states in the wake of economic globalisation (Gifford, 2008; Walby, 1999), i.e. accumulate economic and political capital, just as economic and cultural unification once strengthened the hand of national rulers against regional rivals.

One effect of integration, however, was to restructure the state and stakes of ethno-racial domination in the UK by pairing the old white/non-white opposition with an intensified internal differentiation and hierarchisation of ‘whiteness’ along ethno-national lines. The point of observable transformation in the UK came with enlargement of the EU to incorporate the post-socialist nations of Eastern Europe (Poland, Romania, etc.), the subsequent migrations from East to West in search of comparatively well-paid employment, and the emergence in the UK of a market (shops, foods, etc.) catering to Eastern European tastes. New differences in language, lifestyle and values transpired, giving birth to defensive notions of ‘British culture/values’, allodoxic equivalation of Eastern Europeans with ‘gypsies’ and criminality and evaluative construction of their culture as ‘backward’ (Anderson, 2013). Although classifications and evaluations of difference certainly exhibited racialising features, as Romanians, Hungarians and Poles were, like the Irish and Jews before them, cast as ‘degenerate’ whites (Anderson, 2013; Fox et al., 2012), they nonetheless revolved explicitly around nationality or region (Poles, Eastern Europeans) rather than skin pigmentation, and Eastern Europeans could use their whiteness to their advantage (Fox, 2013; Fox et al., 2014). In fact, Flemmen and Savage (2017) have suggested that the precise emphasis varied by position in the social space, at least among older people: while those rich in cultural capital may have frequently been in favour of multiculturalism, those richer in economic capital were likely to retain vestiges of an ‘imperial racism’ (cf. Dorling & Tomlinson, 2019), but the dominated class were more likely to articulate a proud anti-establishment yet colour-blind form of nationalism separating ‘British born and bred’ from others (i.e. Eastern Europeans).

Meanwhile, dominant players within the institutions of the EU had begun to favour and encourage a specific vision of political economy, articulated in the Social Charter, involving interregional economic redistribution, labour market regulation and institutionalisation of worker rights (Gifford, 2008). Although refracted through various struggles within the fields of agents constituting the EU’s organisations and agencies, the model resembled the regime of the dominant nations – as determined by their place in the field of nation states – within the EU at the time, especially the social market model of West Germany. This alarmed those on the right of the British political field who viewed the Social Charter as a threat to the neoliberal form of deregulation they were steadily imposing (see Evans, 2004; Green, 2006: 181ff). Then the Maastricht Treaty established the unifying strategy of the EU via currency reform and economic regulation, spurring the ‘Maastricht Rebels’ within the ruling Conservative Party to almost topple the Prime Minister at the time, John Major, and the formation of the anti-EU Referendum Party (Sowemimo, 1996). The Lisbon Treaty a decade later continued the unifying strategy by centralising juridical and political capital in the form of legal sovereignty, a powerful parliament and the Presidency. The UK government wrestled certain opt-outs from the legal conditions of the EU (just as it had opted out of the common currency), but its ratification was nevertheless seen as a surrendering of powers by Eurosceptic Conservatives (represented above all by the European Research Group) and others favouring free markets. Ultimately, what started as a strategy for re-ascent in the field of nation states was now perceived as not only a threat to national position *but a threat to existence as an agent in the field*, with many major and minor players of the domestic political field intuiting – and arguing – that the UK state was now being subsumed within a larger entity with control over capital (a federal or ‘United States of Europe’) rather than using it to bolster its own position. Thatcher herself is emblematic of this shifting perception: originally pro-EC (and EU) in the 1970s and early 1980s on account of its potential for aiding free trade, and thus the UK’s economic capital, she decried Maastricht as a ‘treaty too far’ (Gowland & Turner, 2014: 281) and eventually called for fundamental renegotiation of EU membership or else withdrawal (and joining NAFTA) in order to safeguard sovereignty and pursue favourable trade deals (Thatcher, 2002).

## The unintended consequences of deindustrialisation

To summarise the first level of analysis, then, membership of the EEC/EU and deindustrialisation were components of a conservation strategy within the field of nation states, but subsequent transformation of the EU was seen by many political actors to have rebounded on the UK. Deindustrialisation, meanwhile, had other unintended consequences: the mutation of the class structure and the space of political position-takings that has occurred since the 1970s. The major movement in this regard, and often highlighted but less often rigorously analysed, is the numerical decline of the ‘old working class’ – those employed in primary and secondary industries or skilled work, with little cultural capital relative to economic capital – as well as the old petite bourgeoisie, whose remaining members are thus typically older in years (Atkinson, 2017; Evans and Tilley, 2017).

A lack of institutionalised cultural capital, i.e. lower education, has long been associated with a more conservative-traditionalist orientation on identity and culture (Evans, 2000; Heath et al., 1985; Lipset, 1960); in the past, though, this was overridden among the dominated class in Britain by a *collectivist* ethos guiding those with lower cultural capital toward the Labour Party. However, over time, as economic conflicts became less pronounced after deindustrialisation and the dismantling of trade unions, and as non-materialist issues (e.g. gender relations, immigration) became more pronounced with feminisation of the workforce and the diversification of the ethno-racial/national field, the conservatism of those with less cultural capital became more noticeable and, as many have suggested, characteristically backward-looking: the past was better, when women stayed at home and foreigners stayed in their countries, because their conditions were (so they believe) better then – their expectations for the future were only of further decline. On the other hand, there is the rise of class fractions richer in cultural capital: young, liberal, urban and cosmopolitan in their views and with a sense of becoming the new guard. This is the divide that Kriesi et al. (2008, 2012) have described in terms of ‘globalisation winners and losers’.

The relationship between the social space and the political field is dialectical: just as shifts and strategies within the political field engendered transformations of the social space, so those transformations started to feed back into the expectations and strategies within the political field – a sense of what would win followers (the specific stakes of the political field) and elections, that is. Crucial here is of the rise of ‘New’ Labour in the 1990s: in tandem with the decline of the old working class, the proliferation of younger, cultural-capital rich cosmopolitans and the electoral successes of the Conservatives and the Liberals, subversive players within the Labour Party (as a sub-field of the political field) began, with increasing success, to champion a ‘modernising’ agenda, eventually resulting in the abandonment of socialism (Clause 4 of its constitution) and the overt championing of social liberalism and multiculturalism (even if undermined in practice: Back et al., 2002; Squire, 2005; on New Labour’s rise more generally, see Heath et al., 2001). This was no rational calculation in pursuit of the gains of office, but a case of the modernisers championing what they ‘believed in’ – their liberal dispositions in tune with their high cultural capital relative to many members of the Labour Party (they were typically educators, barristers, journalists etc. rather than miners and union officials) – while arguing ‘society [i.e. the social space and associated attitudes] had changed’ and that the Party must reflect that (Blair, 2010: 45). The old working class, as many have observed, thus became marginalised and voiceless – there was no longer an obvious party line they could use to articulate their class ethos (Evans and Tilley, 2017; Atkinson, 2017; Piketty, 2020).

David Cameron, as leader of the Conservative Party, subsequently began to champion social liberalism and ‘compassionate conservatism’ as part of an explicit effort to change *his* party’s image and appeal to young cosmopolitans. This too was born not of utilitarian calculation, but, in part, of his own embodiment of the emergent orthodoxy of his class and the political field: what he ‘believed in’ thanks to his upbringing and education, a sense of what was the ‘right thing’, but also a sense that the Conservatives needed to be the ‘heirs to Blair’ to win elections (Cameron, 2019: 53). Another part came from his sense of the shifting social space and distribution of ethico-political distributions: an awareness that the electorate was ‘more urban and ethnically diverse’ and that ‘social attitudes and customs were changing’ in favour of liberalism and environmentalism (Cameron, 2019: 92). The same marginalisation that afflicted the old working class with Labour’s ‘modernisation’ may thus have affected sections of the upper-working class and petite bourgeoisie traditionally voting Conservative. This is a question of a *shifting homology between the social space and the political field*. The Labour Party no longer corresponded so strongly with the working class, gravitating instead toward the cultural-capital rich sections of the dominant/intermediate classes, and likewise the Conservative Party may have increased its appeal to the capital-rich. The political position-taking that corresponded most strongly with the lower and older working class was one of cynicism, disengagement and disenfranchisement (Atkinson, 2017).

Now the political field, as already mentioned, shares many affinities with the religious field: both are ultimately geared toward the winning of followers through articulation and representation of their interests, i.e. through offering a vision that resonates with an ethos and promises rescue – a laity in one case and an electorate in the other. The main struggle within the religious field is between the established church authorities, with their institutionalised forms of capital, and the newcomers, the prophets offering new visions and aiming to subvert the rules of the game, who through charisma and perceived affinity of habitus manage to win followers (Bourdieu, 1987). The most successful are those who intuit the gaps, the places of weak homology; who perceive a section of the laity whose ethos sits uncomfortably or not at all with currently supplied vision of the world. And so it is the same in the political field. After the failure of the Referendum Party (perceived as too economically liberal) and the British Nationalist Party (perceived as too fascist) to make great inroads, there was UKIP. To draw the analogy between religious prophets and Nigel Farage might seem extreme, but Farage shared many of the hypothesized structural conditions and strategies: a degree of apparent ‘charisma’, for sure, but also perceived social proximity, and thus correspondence, with a broader zone of the social space. He may well have been privately educated, but he did not go to university, separating him from the so-called ‘liberal elite’; he may well have had an affluent background, but he gave the impression of being self-made through stock-broking; his tastes and mannerisms (including accent) were close enough to those of the working class (especially via interest in pubs) but also the economically rich and those who aspire to be like them (sharp suits, expensive dinners, etc.).

Being relatively rich economically, but not rich enough to reach the elite, and not so rich in cultural capital, Farage’s own ethos originally seemed fairly petit-bourgeois (and of course he had great admiration for Thatcher, the archetypal petit-bourgeois conservative), oriented around an anti-EU stance on the grounds of over-regulation stifling accumulation of economic capital. Yet his success appeared to have come in exploiting the possibilities of the political field – perceived by himself and a wider pool of UKIP functionaries – given its shifting homology with class (Clarke et al., 2017; Goodwin & Milazzo, 2015). The marginalisation of the old working class opened up a sizeable region of the social space without a voice, a batch of potential followers who, perhaps, were close enough to Farage’s own ethos to be able to be co-opted with the right articulation but also far enough for economic interests to be at odds. Farage’s populism, anti-liberalism and increasing attention to migration and Islam (well documented as time went on) were tactics in this co-optation strategy. He could appeal to the traditional-conservative element of the old working-class ethos when it game to post-materialist issues, play on their fears given the shifting nature of the ethno-racial/national field (once a compensatory source of security and dominance for the white working class, but seemingly under threat in the post-9/11 era), blame failing health services and limited welfare provision on greedy if not criminal (Eastern European) migrants rather than laisser-faire politics, and so on. All of those could be rhetorically tied back to the EU in one way or another – budget contributions, immigration policy, over-hasty Eastwards expansion, mishandled refugee crises and so on. UKIP’s economic programme, on the other hand, was always on the political right, which could be pitched to the upper working class and petite bourgeoisie as a means to help then ‘get on’, but which was generally de-emphasised because structurally ‘further away’ sections of the working class they were trying to appeal to still held to fairly egalitarian economic principles (and of course at the other end of the scale, UKIP could make broad common cause on Europe with far-left socialists decrying the EU as a capitalist club).

Homologous social transformations across other Western nations produced homologous agents within and position-takings in their respective political fields, of course, including Marine Le Pen in France or Geert Wilders in the Netherlands. What UKIP had that they did not, however – and which may in part explain why there has been Brexit but no Frexit or Nexit (yet) – was appeal to a seemingly glorious imperial past (almost) within living memory and, more particularly, a vision of existing or easily reconnected economic ties with former colonies now on the rise economically, including India, China (via Hong Kong) and Nigeria, that could replace or surpass trade relations with the EU.^9^ In other words, UKIP mobilised a narrative, designed to allay residual fears among some economic agents about trade ties, rooted in perception of the *ongoing mutations of the field of nation states* – not only the declining trajectory of the UK, but the ascendant trajectories of its populous former colonies.

UKIP’s growing success, and their attraction of Conservative followers in particular, agitated far-right members of the Conservative Party who anticipated electoral usurpation. David Cameron, faced with the field of possibles before him, sensed that offering a referendum on EU membership would be a tactic to face down agitation within his party and secure political capital for himself – as is well known, Cameron firmly believed that the referendum would be won by the Remain camp fairly easily (Shipman, 2017; Smith, 2018). Part of the reason it was not successful was because of struggles for position within the intra-party field (i.e. efforts by Boris Johnson and Michael Gove, and their supporters, to undermine Cameron), but everything would appear to indicate that it may also have been a misfire on Cameron’s behalf, a *misreading* of the state of play, an underestimation of the long-term effects of neoliberalism and marginalisation, a misperception of his own liberal cosmopolitanism as more doxic or firmly orthodox than it really was given his social and physical distance from the fallout of thirty years of laisser-faire politics – something which Bourdieu would put under the label of allodoxia, the mistaking of one thing for another due to social distance.

All in all, then, de-industrialisation, immigration and political convergence generated a declining and alienated left-authoritarian class fraction whose ethos no longer gelled with an available party line. UKIP and their allies in the political field intuited the gap and, given their expectations of the state of play, pitched their image and message to appeal in a bid to win broad support despite the mismatch of economic interests. An anti-EU message was grafted on to anti-immigrant and anti-establishment ethos, increasing numbers came to see withdrawal from the EU as a solution to their fears and problems, and in 2016 they had a chance to vote on it. The ultimate result was something Bourdieu detected in a range of fields: a *conservative revolution* – the game was changed forever by those calling for a reversal, a return to the old, a halt to their perceived decline. Joining back up to the previous summary, this national conservative revolution was ultimately the unintended by-product of a conservative strategy in the field of nation states, with the British political field as critical mediator.

## Conclusion

The effects of Brexit are being and will be translated into the specific logic of pertinent fields, which, in the UK, is surely almost all of them. Active careers in the political field have already - for now - come to a close, ending either in failure (Cameron) or victory (Farage), because of the referendum result; withdrawal from EU trade relations and regulations provide new possibilities and impossibilities, and uncertainties, for players within the economic field; exit from agreements on scientific collaboration and student exchange will ricochet through the intellectual field; changing likelihoods for consumption and travel will affect the symbolic translation of class positions; and so on. Even the smallest-scale micro-fields, like the family, have been riven by internal, intergenerational antagonisms on European integration (Davies, 2021). And then there are the possible effects of withdrawal on the precise position of the UK in the field of nation states, though whether it will mark an accelerated decline or be a stabilising force is for time and future research to tell. From the smallest to the largest social structure and many in between, however, the mediating factor will always be, from the perspective adopted here, agents’ changing perceptions of the possible and impossible, the likely and the unlikely and so on; that is, their practical sense embedded in the temporal structures of consciousness and the socially differentiated experiences informing them.

The sociogenetic model sketched out in this paper is open to confirmation and revision in light of further, detailed research on the various historical struggles and strategies within relevant fields. One point worth underscoring, however, is that no amount of intra-field detail will diminish the core conceptual point that the origin of Brexit is a multi-field phenomenon, involving the interplay and intersection of several relatively autonomous structural spaces. This is to extend on Bourdieu’s typical *modus operandum* of situating a target field within the field of power and background alterations in the class structure, and to suggest that there is value, i.e. analytical payoff, in moving between a narrower focus and a wider view of field interrelations so that elements can be identified for more targeted analysis. It is also to recognise that agents are often invested in more than on field, that their strategies are often double or multi-plays and that they have not just a feel for a game but a feel for the *games* they are playing. These include games Bourdieu himself did not consider, such as the ethno-racial field and the field of nation states – though he may well have done had he lived longer – but which others have since posited and which have proven to be crucial pieces of the puzzle of Brexit. That they are likely to be crucial for grasping other trans-field objects of inquiry, from the impact of Covid-19 to Russia’s invasion of Ukraine, seems highly likely.

## Footnotes

1 Wacquant (2020) himself is opposed to the idea of an ethno-racial field, but on what strike me as spurious grounds.

2 The structure of the field anno c. 2016 has been mapped empirically using methods of geometric data analysis in Atkinson (2022).

3 This was a double play, of course, insofar as it also represented a strategy in the British *political* field to present socialism and the Labour Party as progressive and the ‘old boy’ traditionalism of the Conservatives as backwards. The speech text is archived at http://nottspolitics.org/wp-content/uploads/2013/06/Labours-Plan-for-science.pdf.

4 Hansard (17^th^ February 1972).

5 Calculations are based on figures from the World Bank Database (https://data.worldbank.org/).

6 Parallel developments in the US are discussed in Atkinson (2022).

7 Quotes are taken from the texts of Conservative Party Manifestos archived at http://www.conservativemanifesto.com/

8 The EU and its institutions have proved fertile objects of investigation for those finding Bourdieu’s tools of capital and field analytically fruitful, but there is not quite a consensus on how exactly to conceptualise them. See, for example, Georgakakis and Rowell (2013), Kauppi (2005, 2018), Kauppi and Madsen (2013), the special issue symposium of *International Political Sociology* on ‘Bourdieu and the International’ (vol 5, issue 3) and, more recently, Johansson et al., (2021). The statement here attempts to combine the insights.

9 This view was expressed explicitly, for example, by prominent UKIP politician Paul Nuttall in an opinion piece in one of the UK’s prominent right-wing tabloids on the eve of the 2016 referendum (the *Express*, June 22, 2016). There was also a cultural component – an argument about greater cultural similarity to former colonial subjects than Eastern Europeans – aired on television by Farage (BBC, April 22, 2015) and harmonising the economic argument with the appeal to British identity.
